# Combination Pharmacotherapies for Obesity—Current Evidence and Future Directions

**DOI:** 10.1002/edm2.70307

**Published:** 2026-08-11

**Authors:** A. B. M. Kamrul‐Hasan, Arnav Kalra, Nitin Kapoor, Deep Dutta, Kunal Mahajan, Robin Maskey, Syed Abbas Raza

**Affiliations:** ^1^ Department of Endocrinology Mymensingh Medical College Mymensingh Bangladesh; ^2^ Department of Endocrinology & Metabolism All India Institute of Medical Sciences New Delhi India; ^3^ Department of Endocrinology, Diabetes and Metabolism Christian Medical College Vellore Tamil Nadu India; ^4^ Non‐Communicable Disease Unit, Melbourne School of Population and Global Health University of Melbourne Carlton Victoria Australia; ^5^ Department of Endocrinology CEDAR Superspeciality Healthcare New Delhi India; ^6^ Department of Cardiology Himachal Heart Institute Mandi Himachal Pradesh India; ^7^ Department of Internal Medicine B.P. Koirala Institute of Health Sciences Dharan Nepal; ^8^ Department of Endocrinology Shaukat Khanum Memorial Cancer Hospital and Research Centre Lahore Pakistan; ^9^ Department of Endocrinology National Defense Hospital Lahore Pakistan

**Keywords:** combination pharmacotherapy, fixed‐dose combinations, GLP‐1 receptor agonists, multi‐agonist peptides, obesity management medications, precision obesity medicine

## Abstract

**Introduction:**

Conventional single‐agent drugs yield modest, plateauing weight loss and often lead to weight regain. Combination pharmacotherapy is a logical next step to target complementary pathways of weight control while reducing toxicity and treatment burden. This review synthesises mechanisms and clinical evidence for fixed‐dose combinations, multi‐agonist peptides, add‐on regimens and multimodal strategies integrating pharmacotherapy with procedures.

**Methods:**

We conducted a narrative review of clinical trials and observational studies on approved and emerging obesity pharmacotherapy combinations, focusing on mechanistic complementarity, efficacy, safety and practical implementation. Agents were mapped to three domains of energy homeostasis—homeostatic appetite regulation, hedonic‐reward circuitry and peripheral metabolic effector pathways—to inform interpretation of combination strategies.

**Results:**

Fixed‐dose combinations such as phentermine‐topiramate and naltrexone‐bupropion yield greater weight loss than their individual components but also carry gastrointestinal, cardiovascular and neuropsychiatric risks. Multi‐agonist peptides integrating GLP‐1, GIP, glucagon, or amylin deliver double‐digit weight loss and improve glycemic and cardiometabolic profiles, but increase the risk of gastrointestinal adverse events, requiring close monitoring. Add‐on regimens that combine GLP‐1 agonists with approved obesity medications or SGLT‐2 inhibitors, and multimodal strategies that pair drugs with bariatric or endoscopic procedures, generally show additive rather than truly synergistic effects on weight loss.

**Conclusions:**

Available data suggest that combination pharmacotherapy can extend current approaches to precision obesity care, generally providing additive benefits alongside increased regimen complexity, costs and adverse‐event risks. Thoughtful, phenotype‐guided selection and comparative studies versus potent monotherapies, enriched by multi‐omics and behavioural insights, may clarify which patients benefit most and sustainably from these strategies.

## Graphical abstract

Combination pharmacotherapy for obesity can enhance weight loss by targeting complementary neuroendocrine and metabolic pathways. Such benefits are largely additive but limited by tolerability, cost and access. This review outlines practical clinical archetypes and a research agenda for safer, more precise and durable combination strategies across approved, investigational and off‐label regimens.

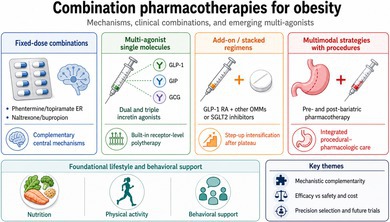

**Abbreviations**
AE: adverse events
BAT: Brown adipose tissue
BMI: body mass index
BP: blood pressure
CVD: cardiovascular disease
EMA: European Medicines Agency
ER: extended‐release
FDC: fixed‐dose combination
GABA: gamma‐aminobutyric acid
GCG: glucagon
GCGR: glucagon receptor
GI: gastrointestinal
GIP: glucose‐dependent insulinotropic polypeptide
GLP‐1: glucagon‐like peptide‐1
HbA1c: glycated haemoglobin
HDL: high‐density lipoprotein
IBT: intensive behavioural therapy
LDL: low‐density lipoprotein
MASH: metabolic dysfunction‐associated steatohepatitis
MASLD: metabolic dysfunction‐associated steatotic liver disease
NB: naltrexone/bupropion
NMPA: National Medical Products Administration
OMMs: obesity management medications
POMC: pro‐opiomelanocortin
RA: receptor agonist
RCT: randomised controlled trial
REDEFINE: CagriSema phase 3 program
RYGB: Roux‐en‐Y gastric bypass
SC: subcutaneous
SCALE: liraglutide intensive behavioural therapy program
SGLT‐2: sodium‐glucose cotransporter‐2
SR: sustained‐release
STEP: semaglutide treatment effect in people with obesity program
SURMOUNT: tirzepatide obesity program
T2D: type 2 diabetes
TPM: topiramate
US FDA: United States Food and Drug Administration
β3: beta‐3 adrenergic receptor

## 1 Introduction

Obesity is a major 21st‐century health challenge, increasing in nearly all regions and age groups. Although some countries have flattened the curve, none have consistently reduced adult obesity nationwide [1]. It is associated with type 2 diabetes (T2D), cardiovascular disease (CVD), metabolic dysfunction‐associated steatotic liver disease (MASLD), certain cancers, osteoarthritis and psychosocial challenges. These issues decrease quality of life and lifespan. Achieving modest weight loss can enhance glycemic control, blood pressure, lipid levels and overall function. However, maintaining weight loss over the long term is difficult without additional interventions beyond lifestyle modifications [2]. Pharmacotherapy has therefore become increasingly central to obesity management. Earlier agents, such as orlistat and sympathomimetics, produced modest weight loss but were limited by gastrointestinal, cardiovascular and neuropsychiatric side effects. Recently, gut hormone–based therapies, such as glucagon‐like peptide‐1 (GLP‐1) receptor agonists (GLP‐1 RAs), have transformed expectations by achieving double‐digit weight loss in some treated patients [3]. A monotherapy approach with approved obesity management medications (OMMs) faces several issues: typical placebo‐subtracted weight loss is 5%–10%, many patients respond poorly, weight loss plateaus and is followed by partial regain, and dose escalation may be limited by tolerability or safety concerns [4].

These observations highlight the complex biological mechanisms of body weight regulation, involving a neuroendocrine network that integrates signals from adipose tissue, gastrointestinal tract, pancreas, liver, hypothalamus, brainstem and cortical areas to control appetite, satiety, food preferences and energy expenditure. Overlapping hormonal pathways, including leptin, insulin, GLP‐1, glucose‐dependent insulinotropic polypeptide (GIP), amylin, ghrelin and peptide YY, act in concert. Weight loss triggers adaptations that increase hunger, reduce satiety, increase thermogenesis and enhance hedonic eating drive [5]. Targeting a single receptor or pathway may not be sufficient to overcome these homeostatic and hedonic mechanisms. Combination pharmacotherapy is a rational approach, and combining agents with distinct mechanisms of action can enhance weight loss. One agent may act as a noradrenergic appetite suppressant, while another may promote satiety or modulate reward‐driven eating. Using lower doses of each can still be effective, leading to greater energy intake reductions and better metabolic outcomes than monotherapy. Fixed‐dose combinations like phentermine/topiramate ER (PHEN/TPM ER) and naltrexone/bupropion (NB) are more effective than placebo for weight loss and often outperform their individual components. Emerging multi‐receptor peptide agonists targeting GLP‐1, GIP and glucagon (GCG) receptors (GCGRs) offer ‘built‐in polypharmacy’ in one molecule, providing notable weight‐loss and cardiometabolic benefits [6].

But enthusiasm for combination strategies must be balanced against concerns about additive adverse effects, increased regimen complexity, higher costs and limited long‐term safety data. This thematic review summarises current evidence on combination pharmacotherapy for obesity and outlines future research directions. Mechanistic approaches to drug combinations are reviewed, data on approved fixed‐dose and other dual‐drug regimens are compiled, emerging multi‐agonist peptides are highlighted, and a research agenda is proposed to identify safe, effective and cost‐effective combination strategies for obesity management.

## 2 Conceptual Frameworks for Combination Pharmacotherapy

Conceptualising combination pharmacotherapy for obesity in terms of discrete physiological domains of energy homeostasis—such as homeostatic appetite regulation, hedonic/reward processing and peripheral metabolic effector pathways—provides a structured framework for rational regimen design. By mapping individual agents to their principal sites of action and modes of action within these domains, and selecting combinations that engage complementary rather than redundant mechanisms, clinicians and investigators can move beyond a descriptive listing of drug pairs toward a mechanistically informed strategy that maximises efficacy while constraining toxicity and treatment burden.

### 2.1 Mechanistic Axes for Combination

One mechanistic axis consists of central appetite and satiety regulators. Noradrenergic–dopaminergic sympathomimetics such as PHEN primarily suppress hunger via hypothalamic and brainstem pathways. TPM modulates GABAergic and glutamatergic signalling. This modulation decreases appetite and may attenuate food reward. The NB combination boosts proopiomelanocortin (POMC) activity and blocks autoinhibitory opioid feedback; these actions increase satiety and reduce cravings [7, 8, 9]. GLP‐1 RAs also play a key role by acting on brainstem and hypothalamic nuclei. They reduce appetite, delay gastric emptying and improve glycemic control [10].

A second axis comprises peripheral/metabolic targets. These include pancreatic and renal mechanisms, as well as gastrointestinal and adipose tissue effects. Orlistat acts in the gut lumen by inhibiting pancreatic lipase, thereby reducing fat absorption and, secondarily, energy intake [11]. Sodium‐glucose cotransporter‐2 (SGLT2) inhibitors, although primarily developed for diabetes, induce a negative energy balance by promoting urinary glucose excretion and modest weight loss [12]. GCGR agonists, in isolation or combined with GLP‐1 agonism, can increase energy expenditure and promote lipid oxidation. Agents that activate brown adipose tissue or beige adipocyte thermogenesis (such as certain experimental β3‐adrenergic agonists or GCG‐containing peptides) aim to increase resting energy expenditure [10]. These peripherally acting drugs are attractive partners for centrally acting agents because they can enhance the metabolic efficiency of weight loss without further intensifying central appetite suppression.

**FIGURE 1:**
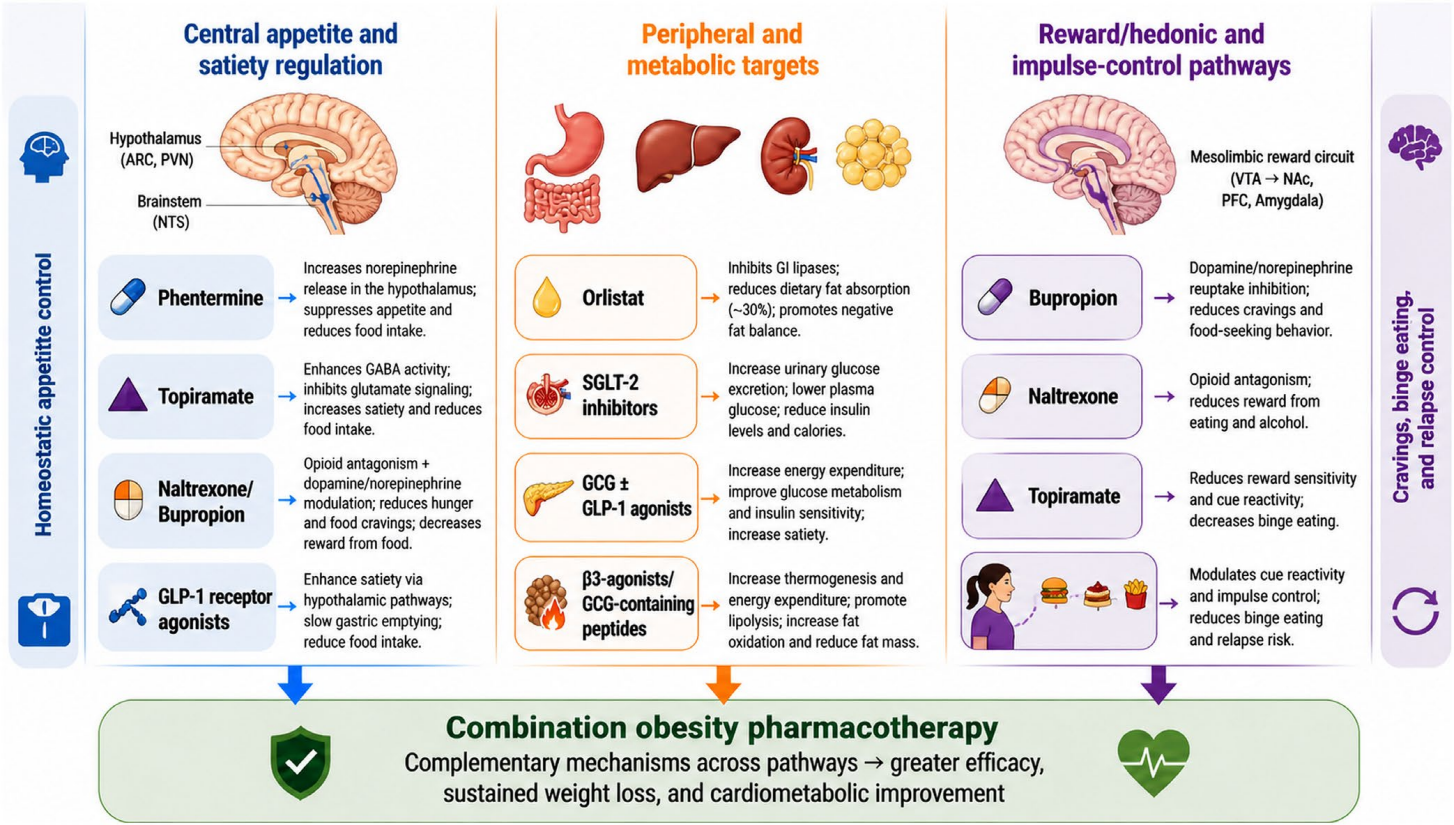
Conceptual framework for combination obesity pharmacotherapy, mapping centrally acting agents, peripheral/metabolic targets and reward/hedonic pathway modulators to complementary mechanisms that reduce energy intake, enhance energy expenditure and support sustained weight loss with cardiometabolic improvement. ARC, arcuate nucleus; GABA, gamma‐aminobutyric acid; GCG, glucagon; GI, gastrointestinal; GLP‐1, glucagon‐like peptide‐1; NAc, nucleus accumbens; NTS, nucleus tractus solitarius; PFC, prefrontal cortex; PVN, paraventricular nucleus; SGLT‐2, sodium‐glucose cotransporter‐2; VTA, ventral tegmental area.

A third axis involves reward/hedonic and impulse‐control pathways. Corticolimbic circuits, including dopaminergic pathways in the ventral striatum and prefrontal regulatory regions, mediate hedonic overeating and food‐addiction‐like behaviours [13]. Bupropion and naltrexone, by modulating dopaminergic and opioidergic signalling, appear particularly relevant for patients with high cravings, binge‐eating tendencies, or strong cue‐driven intake [9]. TPM, via GABA/glutamate modulation, also has evidence supporting its use to reduce binge‐eating behaviour [8]. Conceptually, agents targeting this hedonic axis may be especially useful when combined with drugs that predominantly affect homeostatic appetite control to blunt relapse driven by stress, cues, or emotional eating. These three mechanistic domains and representative agents are summarised schematically in Figure 1. These conceptual archetypes set the stage for currently approved clinical combinations and other two‐drug regimens, which are discussed in the next section.

### 2.2 Types of Combination Strategies

Within these mechanistic axes, several broad strategic archetypes for combination pharmacotherapy can be defined. The first is fixed‐dose combinations with complementary mechanisms. PHEN/TPM ER pairs a sympathomimetic with a neuromodulator to deliver greater weight loss than either component alone at the same doses, with evidence of improved durability [8]. NB similarly combines a dopaminergic–noradrenergic antidepressant with an opioid receptor antagonist to augment POMC‐mediated satiety and dampen reward‐driven intake [9]. Bupropion/zonisamide, although less widely used, follows a similar logic: combining a monoaminergic agent with a neuromodulator that reduces appetite and may influence food reward [14].

The second archetype is the multi‐agonist single molecule, which embodies pharmacologic polytherapy within a single peptide or small molecule. Dual incretin agonists (GLP‐1/GIP) and triple agonists (GLP‐1/GIP/GCG) combine actions across multiple receptors that influence appetite, insulin secretion, lipid metabolism and energy expenditure. GLP‐1/GCG co‐agonists aim to pair the anorectic and glucose‐lowering effects of GLP‐1 with the thermogenic and lipid‐oxidising properties of GCG, whereas GLP‐1/amylin combinations seek to leverage complementary satiety and gastric‐emptying effects [10]. Although clinically administered as single agents, these molecules functionally recapitulate the logic of combination pharmacotherapy at the receptor level, with the added advantages of simplified dosing and potentially more predictable pharmacokinetics.

A third archetype is add‐on or step‐up strategies using separately prescribed drugs. Examples include adding a GLP‐1 RA to ongoing OMM in patients who initially respond but then plateau, or combining a GLP‐1 RA with an SGLT2 inhibitor in individuals with type 2 diabetes (T2D), where both weight loss and cardiometabolic risk reduction are dual goals. In these settings, the aim is typically to build on the partial efficacy of a first agent, either by complementing its mechanism (e.g., central appetite suppression plus hormonal satiety) or by adding a distinct metabolic effect (e.g., urinary glucose loss) [15]. Clinically, this can be implemented as an early combination in high‐risk phenotypes or as stepwise intensification once a suboptimal response is evident.

A fourth archetype extends these pharmacologic approaches to multimodal strategies that include procedures. In this model, obesity medications are integrated with bariatric or endoscopic interventions to enhance the durability of weight loss, address post‐procedural weight regain, or support peri‐procedural optimisation.

**FIGURE 2:**
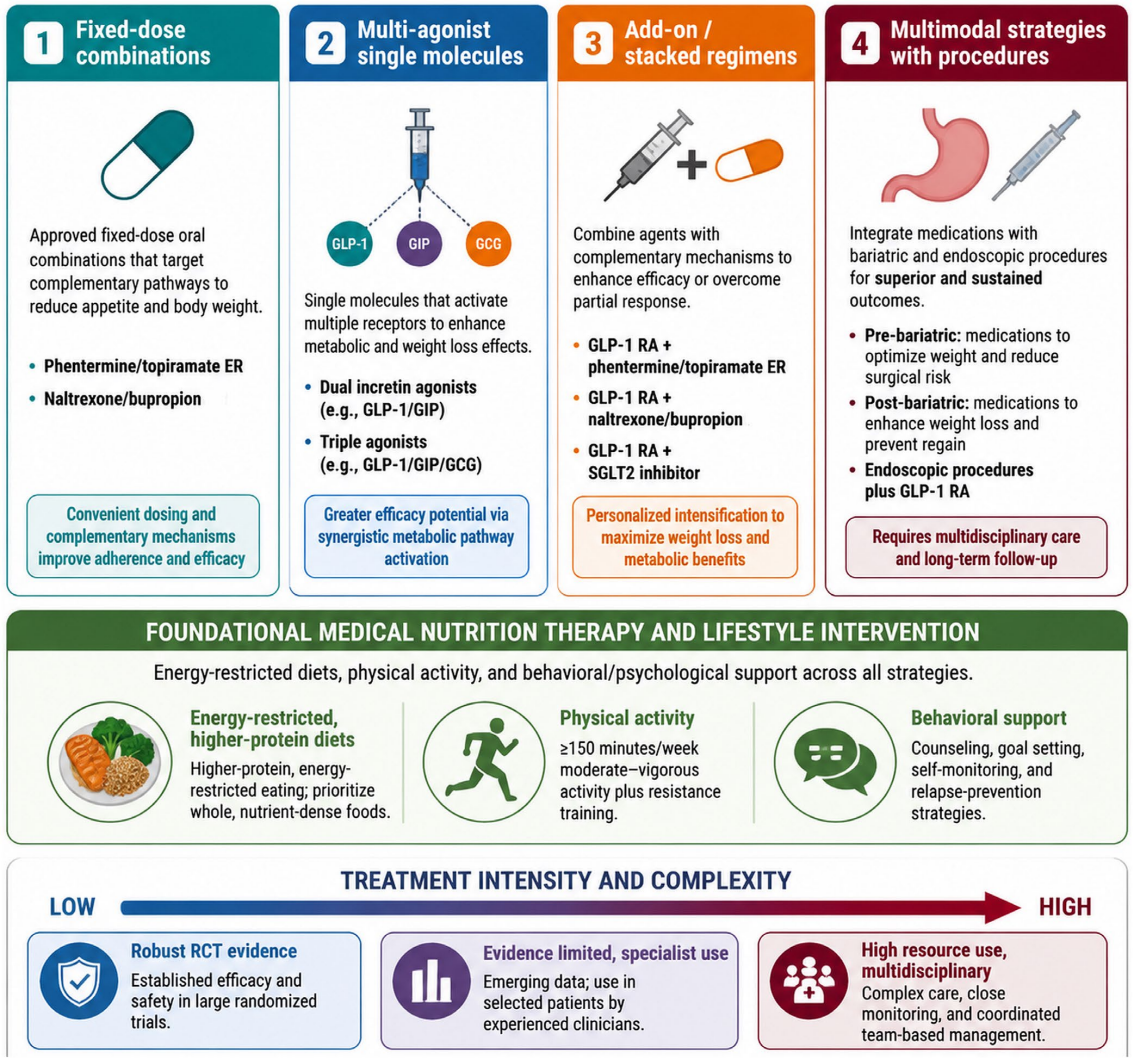
Clinical archetypes of combination pharmacotherapy for obesity. The four panels illustrate fixed‐dose oral combinations, single‐molecule multi‐agonists, add‐on/stacked GLP‐1–based regimens, and multimodal strategies that integrate pharmacotherapy with bariatric or endoscopic procedures, arranged along a continuum of treatment intensity and complexity. All pharmacologic approaches are implemented on a foundation of medical nutrition therapy and lifestyle intervention, typically emphasising energy restriction, higher protein intake, physical activity and behavioural support. ER, extended‐release; GCG, glucagon; GIP, glucose‐dependent insulinotropic polypeptide; GLP‐1, glucagon‐like peptide‐1; RA, receptor agonist; RCT, randomised controlled trial; SGLT2, sodium–glucose cotransporter‐2.

Across all four archetypes, pharmacologic treatment is best viewed as delivered on a common foundation of medical nutrition therapy and lifestyle interventions, including energy restriction, higher‐protein dietary patterns, physical activity and behavioural or psychological support. These pharmacologic archetypes and this shared lifestyle and nutrition foundation are summarised in Figure 2.

### 2.3 Efficacy Versus Safety and the Question of Synergy

Central to any framework for combination pharmacotherapy is the balance between efficacy and safety. In principle, combining agents might yield additive, more‐than‐additive (synergistic), or sub‐additive (redundant or antagonistic) effects on weight and metabolic outcomes. Preclinical studies of leptin plus amylin and of amylin plus GLP‐1 RAs have shown striking synergy: doses that are minimally effective when given alone can produce robust weight loss when co‐administered, accompanied by coordinated changes in hypothalamic signalling pathways [16]. However, in human trials, most combinations evaluated to date have produced effects best described as additive or modestly greater than additive, and some theoretically attractive pairings have failed to deliver a clinically meaningful incremental benefit [17].

At the same time, the adverse‐effect burden of combining agents is likely to be at least additive. Overlapping toxicities, including gastrointestinal effects (e.g., nausea, vomiting), cardiovascular effects (e.g., heart‐rate or blood‐pressure increases), neuropsychiatric symptoms and rare but serious events such as gallbladder disease or pancreatitis, can accumulate across drugs. Polypharmacy also increases regimen complexity, pill or injection burden and cost, with implications for adherence and health‐system affordability [18]. An effective conceptual framework must therefore treat increased efficacy and increased risk as linked variables rather than independent attributes: a combination regimen is justifiable only if the incremental benefit in weight and cardiometabolic outcomes clearly outweighs the incremental risks and costs in the specific patient populations and treatment durations under consideration.

## 3 Established Clinical Combinations

In practice, multiple fixed‐dose and two‐drug combinations already have trial data and regulatory approval. Fixed‐dose combinations (FDCs) and other extensively studied two‐drug regimens stand as the most developed forms of combination pharmacotherapy for obesity. They provide proof of principle that targeting complementary mechanisms can yield greater weight loss than monotherapy, while also illustrating the practical constraints imposed by safety, tolerability and regulatory requirements. Among these, PHEN/TPM ER and NB are the best characterised in terms of randomised controlled trial (RCT) evidence and real‐world use.

### 3.1 FDA/EMA‐Approved Fixed‐Dose Combinations

#### 3.1.1 Phentermine/Topiramate Extended‐Release

PHEN/TPM ER combines two agents with complementary central effects on appetite and food reward. PHEN primarily reduces hunger through hypothalamic and brainstem pathways, whereas TPM is thought to decrease appetite, attenuate food reward and possibly reduce binge‐eating behaviour. The FDC is designed to improve efficacy while reducing dose‐related adverse events by combining these components at lower doses than are typically used in monotherapy [8]. In the phase 3 EQUIP and CONQUER trials, PHEN/TPM ER produced substantial incremental weight loss compared with placebo, with mean placebo‐subtracted reductions of approximately 7%–9% of initial body weight at the highest approved dose over 52 weeks. A large proportion of participants (often > 50%) achieved at least 10% total body weight loss, and sustained reductions were observed in extended follow‐up for responders maintained on therapy. Blood pressure, triglycerides, HDL cholesterol and glycemic parameters also improved, including a reduced risk of developing T2D in at‐risk individuals [8, 19].

The safety profile reflects the known pharmacological effects of the components of PHEN/TPM ER. Common adverse events include paraesthesia, dry mouth, constipation, dysgeusia and insomnia. Heart rate increases are dose‐related. Cognitive symptoms, concentration and memory impairment have been observed, in agreement with reports from epilepsy patients treated with TPM. Because of the teratogenic risk, particularly the association between first‐trimester TPM exposure and oral clefts, a Risk Evaluation and Mitigation Strategy was developed in the United States. This includes pregnancy testing and contraceptive counselling for women of childbearing potential. Thus, PHEN/TPM ER is usually effective when used with carefully chosen combinations but needs close monitoring for cardiovascular, neuropsychiatric and reproductive safety [8, 19].

#### 3.1.2 Naltrexone/Bupropion

The NB FDC acts via another pathway, through the effect on hypothalamic POMC neurons and the corticolimbic reward system. Bupropion increases POMC activity and alpha‐melanocyte‐stimulating hormone release, thereby promoting satiety via dopaminergic and noradrenergic pathways. At the same time, naltrexone blocks the autoinhibitory opioid feedback to POMC neurons, thereby reducing opioid‐driven reward responses. Together, these effects strengthen natural satiety signals and reduce the likelihood of pleasure‐, craving‐, or cue‐driven overeating [9]. In COR‐I, COR‐II and related phase 3 RCTs, NB produced a mean placebo‐subtracted weight loss of approximately 4%–5% at 1 year, with higher proportions of patients achieving clinically meaningful thresholds (≥ 5% and ≥ 10%) than placebo. Additional benefits included modest improvements in HbA1c, fasting glucose, triglycerides and HDL cholesterol, particularly among participants with T2D or prediabetes. Some trials of NB combined with intensive behavioural interventions suggested that pharmacologic modulation of appetite and reward can enhance the durability of lifestyle‐induced weight loss [9, 20].

Gastrointestinal adverse events, nausea in particular, are common with NB and often dose‐limiting. Elevations in blood pressure and heart rate have been noted, so it should not be used in uncontrolled hypertension, and caution is advised for patients with cardiovascular disease. Bupropion has a dose‐dependent risk of seizures, and doses need to be carefully titrated in those with risk factors. It may also cause neuropsychiatric effects like anxiety, insomnia and mood changes due to its antidepressant action and naltrexone's role in reward processing. These considerations underscore that combinations targeting reward and impulse‐control pathways require particularly careful psychiatric and cardiovascular monitoring [9, 20].

Key efficacy, safety and regulatory features of approved fixed‐dose combinations are summarised in Table 1. These results are derived from phase 3 RCTs in adults with obesity or overweight and may not fully generalise to broader, more heterogeneous clinical populations.

**TABLE 1:** Fixed‐dose combination obesity management medications with regulatory approval: key clinical outcomes.

Combination (components)	Primary mechanisms	Mean placebo‐subtracted weight loss	Major adverse events	Regulatory status/notes
Phentermine/topiramate ER [8, 19]	*Phentermine*: noradrenergic‚ dopaminergic sympathomimetic, hunger via hypothalamus/brainstem; *Topiramate*: GABA/glutamate modulation, appetite and food reward	~7%–9% of initial body weight at highest approved dose in large phase 3 trials in ~1 year	Paresthesia, dry mouth, constipation, insomnia, dysgeusia‚ increased heart rate, cognitive symptoms (attention, memory), teratogenic risk (oral clefts)	Approved for chronic weight management by US FDA and approved in some other countries; not approved for obesity by EMA; requires pregnancy prevention and monitoring for cardiovascular and neuropsychiatric AEs
Naltrexone/bupropion SR [9, 20]	*Bupropion*: dopaminergic/noradrenergic POMC activation; *Naltrexone*: opioid receptor antagonism blocking POMC feedback and reward signalling; net‚ satiety, cravings	~4%–5% of initial body weight at in ~1 year	Nausea, vomiting, headache, dizziness, insomnia, anxiety, blood pressure and heart rate; seizure risk in predisposed patients, neuropsychiatric effects	Approved by US FDA, EMA and in several other regions for chronic weight management; contraindicated in uncontrolled hypertension, seizure disorders and certain psychiatric/withdrawal states
Tirzepatide [21]	Dual GLP‐1/GIP receptor agonist	SURMOUNT‐1: about −11.9%, −16.4% and −17.8% with 5, 10 and 15 mg, respectively; SURMOUNT‐3: an additional −20.8% body‐weight reduction versus placebo after lifestyle run‐in; and SURMOUNT‐4: a −19.4% difference versus placebo favouring continued treatment over withdrawal.	Predominantly gastrointestinal (nausea, vomiting, diarrhoea), usually dose‐dependent and mitigated by titration; modest increases in heart rate; small rises in amylase/lipase; treatment discontinuation due to AEs = 6% at 15 mg in SURMOUNT‐1	Approved for chronic weight management by the US FDA. Also approved in multiple other jurisdictions for adults with obesity or overweight with weight‐related comorbidity based on the SURMOUNT program
Mazdutide [22]	Dual GLP‐1/GCG receptor agonist	Phase 2 trial: −7.7%, −11.4% and −12.3% with 3 mg, 4.5 mg and 6 mg, respectively at 24 weeks; GLORY‐1: −11.3% and −14.31% with 4 mg and 6 mg, respectively at 48 weeks; and 9‐mg obesity trial: −15.4% at 24 weeks	Predominantly gastrointestinal (nausea, vomiting, diarrhoea), mild to moderate. Treatment discontinuation due to AEs = 0.5% at 6 mg in GLORY‐1	Approved in China by the NMPA for chronic weight management based on the GLORY program

Abbreviations: AE, adverse events; EMA, European Medicines Agency; ER, extended‐release; GABA, gamma‐aminobutyric acid; GCG, glucagon; GIP, glucose‐dependent insulinotropic polypeptide; GLP‐1, glucagon‐like peptide‐1; NMPA, National Medical Products Administration; POMC, pro‐opiomelanocortin; SR, sustained‐release; US FDA, United States Food and Drug Administration.

### 3.2 Other Unapproved or Off‐Label FDCs

Outside the United States and the European Union, contemporary access to fixed‐dose OMM combinations largely mirrors that of major Western markets, and no additional PHEN/TPM‐ or NB‐based FDCs have received regulatory approval for obesity. Bupropion/zonisamide was evaluated in phase 2 trials and demonstrated greater absolute weight loss than either component alone or placebo (e.g., ∼9%–10% vs. substantially less with monotherapies and placebo over 24 weeks), supporting the concept that combining a monoaminergic antidepressant with a neuromodulator can enhance appetite suppression and potentially reduce hedonic eating, a concept analogous to those of NB and PHEN/TPM [23]. Development was discontinued, in part because of concerns about zonisamide‐associated cognitive and psychiatric adverse events and the need for more extensive long‐term safety data, and the combination has not progressed to regulatory approval for obesity [24]. In contrast, some countries continue to rely on older or off‐label combinations (e.g., low‐dose sympathomimetic agents co‐prescribed with other drugs) in clinical practice, even though many such regimens lack robust contemporary trial data and should be used cautiously [25].

### 3.3 Other Clinical Two‐Drug Combinations With Human Data

Beyond FDCs, a variety of two‐drug regimens have been studied with separately prescribed agents. These combinations often stem from mechanistic hypotheses or clinical pragmatism, and the evidence base ranges from small RCTs to observational series and case reports.

#### 3.3.1 Bupropion Plus Zonisamide Given Separately

In a short‐term, open‐label, preliminary trial (*n* = 18), the combined administration of zonisamide and bupropion produced greater weight loss than zonisamide alone; over 12 weeks, the combination group lost 7.5% of initial body weight (absolute change) compared with 3.1% in the zonisamide‐only group. Similar to the bupropion/zonisamide FDC, this strategy was not pursued beyond early‐phase trials due to safety concerns [14, 24].

The available data on these off‐label FDCs and two‐drug regimens come primarily from small randomised trials and observational series and should be considered preliminary. Given these limitations, such off‐label combinations are best reserved for specialist obesity or endocrinology practice, with individualised risk–benefit assessment and close monitoring, rather than for routine use in general clinical settings.

#### 3.3.2 Pramlintide‐Based Combinations

Pramlintide, an amylin analog, reduces postprandial glucose excursions, slows gastric emptying and enhances satiety, making it a logical partner for agents that primarily suppress appetite through central pathways. Early trials of pramlintide combined with PHEN or leptin suggested more‐than‐additive weight loss, with some studies reporting double‐digit percentage reductions in body weight among subsets of participants [26, 27]. Mechanistically, these combinations may exploit convergent signalling in hypothalamic and brainstem nuclei while providing complementary effects on gastric emptying and meal‐related satiety. Despite promising early data, development of some pramlintide‐based combinations was curtailed by practical and commercial considerations, including the need for multiple daily injections, gastrointestinal side effects and the difficulty of demonstrating clear superiority over emerging GLP‐1–based therapies in large‐scale trials. Nonetheless, these studies remain important proof‐of‐concept examples of how mechanistically aligned peptide‐based combinations can yield robust effects [28].

### 3.4 Lessons From Current Combinations

Taken together, experience with established combinations yields several practical lessons for the development and deployment of future regimens. First, mechanistic complementarity, such as pairing noradrenergic appetite suppression with neuromodulation or combining POMC activation with opioid blockade, translates into greater average weight loss than monotherapy. Still, effect sizes are constrained by tolerability and safety limits [8, 9, 19, 20]. Second, combinations that engage reward and hedonic circuits may be particularly valuable for patients with high cravings, binge‐eating tendencies, or strong cue‐driven intake. Yet, they also require careful psychiatric and cardiovascular assessment [9]. Third, real‐world use of effective FDCs depends on regulatory rules, monitoring and cost; efficacy in RCTs is a necessary but insufficient condition for broad impact [29]. Ultimately, the disparate outcomes of pramlintide‐based and other experimental combinations underscore the fact that not all mechanistically promising combinations translate into effective or commercially viable therapies. This emphasises the need for rigorous trial design and realistic assessment of adherence and safety in managing complex long‐term obesity treatments [26, 27, 28].

## 4 Combinations With Lifestyle or Procedural Interventions

Beyond pharmacologic pairings alone, OMMs are almost always used alongside lifestyle changes, and recent research indicates they can also serve as pharmacologic ‘scaffolding’ for endoscopic and surgical procedures. These combined approaches generally lead to greater and more sustained weight loss than lifestyle modifications or procedures alone. Evidence in this area is largely based on RCTs, supplemented by observational studies, with limited long‐term follow‐up. However, these approaches also raise important questions about their optimal timing, duration, cost and long‐term safety.

### 4.1 With Structured Lifestyle and Intensive Behavioural Interventions

Across pivotal programs, all currently approved long‐term OMMs have been evaluated on a background of calorie restriction, increased physical activity and some form of behavioural counselling, with consistent incremental benefits over lifestyle alone. In the EQUIP and CONQUER trials of PHEN/TPM ER and COR, combined with dietary and counselling interventions [8, 9, 19, 20]. Orlistat has repeatedly been tested as an adjunct to structured behavioural weight‐loss programs in adults and adolescents; in these studies, orlistat plus behavioural therapy or diet produced modest additional reductions in body weight or BMI compared with behavioural programs alone, with larger effects in individuals without binge‐eating pathology but at the cost of gastrointestinal adverse events [30, 31].

More intensive models have evaluated OMMs specifically as adjuncts to high‐contact behavioural therapy. In the COR‐BMOD trial, NB combined with a 56‐week, high‐intensity group behaviour modification program led to a mean weight loss of about 9.3%, compared with 5.1% with behaviour modification plus placebo, and substantially more participants achieved ≥ 5% and ≥ 10% weight loss [32]. Two trials in primary care and specialty settings show that adding liraglutide 3.0 mg to intensive behavioural therapy results in greater weight loss than therapy alone. SCALE IBT reports a 7.5% loss at 56 weeks, versus 4%; and previous studies also found higher rates of meaningful weight reduction, supporting GLP‐1 pharmacotherapy as an effective booster for behavioural programs [33, 34]. STEP 3 showed that adding semaglutide 2.4 mg once weekly to an initial low‐calorie diet and intensive behavioural therapy led to an average weight loss of 16.0% at 68 weeks, compared with 5.7% with placebo; 86.6% versus 47.6% achieved at least 5% weight loss [35].

More recently, adding mid‐dose PHEN/TPM ER to a digitally enhanced lifestyle intervention (combining group visits, telehealth and app‐based support) resulted in about 9 kg of weight loss over 12 months, compared with roughly 4 kg with the lifestyle program plus placebo [36]. Collectively, these data support the view that approved OMMs work best as pharmacologic amplifiers of lifestyle and behavioural therapy, helping to attenuate compensatory increases in hunger and hedonic drive that otherwise undermine long‐term adherence and weight‐loss durability.

### 4.2 With Endoscopic and Surgical Interventions

Pharmacotherapy is also increasingly being combined with endoscopic devices and bariatric surgery, although the evidence base remains relatively small and heterogeneous. Small trials and observational studies of liraglutide added to intragastric balloon therapy suggest that combining mechanical gastric restriction with GLP‐1 receptor agonism yields greater short‐term weight loss and more favourable body‐composition changes than balloon alone. Adding liraglutide 0.6 mg/day to intragastric balloon therapy led to greater short‐term reductions in weight (13.8 kg vs. 7.9 kg), BMI and body fat, with no new safety issues, supporting a complementary role for GLP‐1 receptor agonism with endoscopic restriction [37]. However, another retrospective study found that adding liraglutide to intragastric balloon therapy did not reduce the risk of weight regain 6 months after balloon removal [38].

In the post‐bariatric setting, pharmacologic treatment of insufficient weight loss or weight regain has focused mainly on GLP‐1 RAs and, to a lesser extent, sympathomimetic/TPM‐based regimens. A 56‐week randomised, placebo‐controlled trial of liraglutide 3.0 mg in adults several years after Roux‐en‐Y gastric bypass (RYGB) demonstrated that liraglutide produced about 10% additional absolute weight loss relative to baseline, whereas those on placebo experienced further weight regain, effectively reversing a substantial portion of weight recurrence without a major increase in serious adverse events [39]. Retrospective cohorts and a recent meta‐analysis suggest that liraglutide and semaglutide typically induce a further absolute weight loss of roughly 5%–13% over 6–12 months in patients with post‐surgical weight regain, with semaglutide often achieving larger reductions than liraglutide, although these comparisons are indirect and unrandomised [40, 41].

In a large retrospective RYGB cohort, use of PHEN and/or TPM—either alone or in combination—significantly reduced cumulative and rapid weight regain relative to nadir weight, indicating these agents can effectively mitigate post‐gastric bypass weight relapse [42]. A dedicated phase 2 study comparing adjunctive PHEN/TPM with placebo in adolescents and young adults (approximately 12–24 years) after bariatric surgery found that PHEN/TPM was associated with a 4.72% greater body weight loss and a 3.61% reduction in BMI. In contrast, participants on placebo experienced weight gain of 0.83% and a 1.89% increase in BMI [43]. The use of NB, TPM alone and off‐label agents (e.g., metformin and SGLT2 inhibitors in people with diabetes) for post‐surgical suboptimal weight trajectories is also reported, although supporting data are sparse and largely observational [44].

## 5 Emerging and Experimental Pharmacologic Combinations

While current combinations provide proof of concept, obesity pharmacotherapy is evolving beyond fixed‐dose products, with ‘built‐in’ molecular combinations and multi‐drug regimens. Traditional combinations use two agents with complementary mechanisms, while newer strategies include multi‐receptor peptide agonism, gut‐hormone‐based add‐ons and hormonal combinations that mimic post‐bariatric or evolutionary energy‐balance profiles. These approaches promise bariatric‐like efficacy but raise concerns about safety, durability and access [3, 6].

### 5.1 Multi‐Receptor Peptide Agonists

Multi‐receptor peptide agonists are the most clinically advanced form of emerging ‘combination’ therapy in obesity. By engineering a single peptide to activate two or more receptors relevant to energy balance, these agents can emulate and in some cases surpass, the efficacy of stacking separate drugs while simplifying dosing and pharmacokinetics. Conceptually, they sit between classic fixed‐dose combinations and monoreceptor agonists: they are single agents from a regulatory and adherence perspective but act as pharmacologic polytherapy at the receptor level [45].

Dual incretin and related multi‐receptor agonists, such as GLP‐1/GIP and GLP‐1/GCG co‐agonists, exemplify this approach. Rather than relying on GLP‐1 receptor agonism alone, they combine complementary incretin and glucagon pathways within a single molecule to influence appetite, insulin secretion, lipid metabolism and energy expenditure [45]. This broader engagement of nutrient‐sensing circuits in pancreatic islets, the hypothalamus and peripheral tissues can yield greater weight loss and metabolic improvement than GLP‐1 monotherapy [45]. Triple agonists that bind GLP‐1, GIP and GCGRs extend the same logic, using carefully titrated glucagon agonism to add thermogenic and lipid‐oxidising effects while GLP‐1 and GIP signalling counterbalance hyperglycemic potential [45]. Early trials report total body weight loss approaching levels seen with metabolic surgery, suggesting that simultaneous modulation of appetite, glycaemia and energy expenditure may achieve efficacy not easily matched by sequential single‐receptor therapies [45].

GLP‐1/amylin combinations, whether as co‐formulations or as unimolecular dual‐function agonists, aim to enhance satiety and slow gastric emptying, thereby stabilising post‐prandial glycaemia and reducing overall caloric intake [46]. Amylin/leptin combinations, though still in development, are supported by preclinical data indicating that synergistic hypothalamic activation can help overcome leptin resistance and produce substantial weight loss in rodent models [47]. Collectively, these developments point toward a future in which receptor‐level multi‐activation, or carefully selected pharmacologic ‘stacks’, plays a central role in obesity treatment, rather than reliance on single‐pathway agents.

#### 5.1.1 Approved Dual‐Agonists

Tirzepatide, a once‐weekly dual GIP/GLP‐1 receptor agonist, is the most clinically advanced ‘twincretin’ and has been approved for chronic weight management in several jurisdictions, based on large phase 3 trials showing mean weight losses exceeding those of established GLP‐1 RAs in people with obesity, with or without T2D. It delivers substantial weight reduction and marked improvements in glycemic control and cardiometabolic risk factors, although gastrointestinal adverse events remain common and long‐term safety surveillance is ongoing [21].

Mazdutide, a once‐weekly GLP‐1/GCGR co‐agonist recently approved in China for obesity or overweight, has shown double‐digit absolute percentage weight loss and favourable effects on hepatic steatosis and cardiometabolic parameters in phase 3 studies. By integrating GLP‐1‐mediated satiety with glucagon‐driven increases in energy expenditure, mazdutide demonstrates that dual agonism can approach the efficacy of more complex multi‐agonist constructs while maintaining a relatively simple dosing schedule. Table 1 summarises the weight‐loss potency, common adverse effects and regulatory approval status of tirzepatide and mazdutide [22].

#### 5.1.2 Multi‐Receptor Agonists Under Development

Several multi‐receptor peptides are in development, combining GLP‐1 with GCG, GLP‐1 with amylin analogs, or amylin with leptin and related adipokines. Retatrutide, a triple GLP‐1/GIP/GCG agonist, has produced unprecedented phase 2 weight loss (−22.1% placebo‐adjusted mean weight loss with 12 mg at 48 weeks) and is now in phase 3 TRIUMPH/TRANSCEND trials [48]. Multiple GLP‐1/GCG co‐agonists (survodutide, pemvidutide) achieve ~12%–13% placebo‐adjusted weight loss with improvements in glycaemia, lipids and liver fat [49, 50]. Maridebart cafraglutide (MariTide), a long‐acting GLP‐1 RA conjugated to a GIP receptor antagonist, has shown 11.1% to 13.8% placebo‐adjusted weight loss at 52 weeks with once‐monthly dosing in phase 2 trials [51]. GLP‐1–amylin approaches include the fixed‐dose cagrilintide–semaglutide combination (CagriSema), which in REDEFINE 1 produced approximately 17.4% placebo‐adjusted total body weight loss at 68 weeks, exceeding semaglutide alone at similar doses [52]. The unimolecular GLP‐1/amylin agonist subcutaneous Zenagamtide (Amycretin) has shown placebo‐adjusted weight loss of 11.7%–23.9%, depending on dose and duration (1.25–60 mg over 20–36 weeks) in a phase 2 trial [53].

**FIGURE 3:**
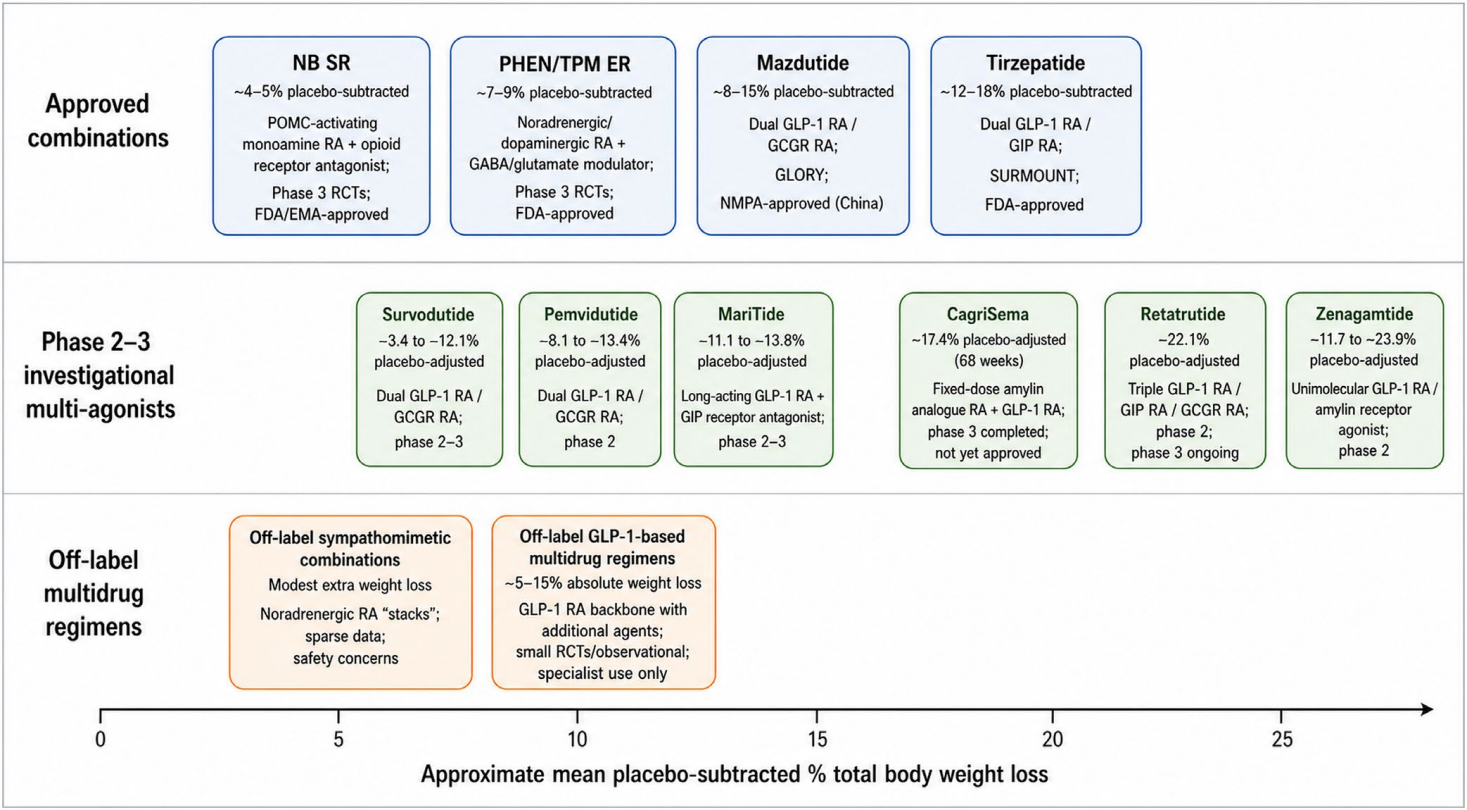
Summary of approved fixed‐dose combinations, approved and investigational multi‐receptor agonists, and off‐label multidrug regimens for obesity, positioned by approximate mean placebo‐subtracted total body weight loss and grouped by evidence level and regulatory status. EMA, European Medicines Agency; ER, extended‐release; FDA, United States Food and Drug Administration; GCG, glucagon; GCGR, glucagon receptor; GIP, glucose‐dependent insulinotropic polypeptide; GLP‐1, glucagon‐like peptide‐1; NB, naltrexone/bupropion; NMPA, National Medical Products Administration; PHEN, phentermine; POMC, proopiomelanocortin; RA, receptor agonist; RCT, randomised controlled trial; SR, sustained‐release; TPM, topiramate.

Together, these agents may deliver bariatric‐range efficacy while preserving a pharmacologic route. However, current evidence remains limited to early‐phase studies, and long‐term safety, durability and comparative effectiveness remain uncertain. The pramlintide–metreleptin amylin–leptin combination, although demonstrating ~12%–13% weight loss and clear amylin–leptin synergy in a proof‐of‐concept human trial, has been discontinued due to safety, immunogenicity and strategic considerations [26]. It remains an important mechanistic prototype rather than an active development candidate. Key efficacy, metabolic, safety and development‐phase features of multi‐receptor agonists under development are summarised in Table S1. The spectrum of efficacy and evidence quality, ranging from approved combinations to off‐label multidrug ‘stacks’, is illustrated in Figure 3.

A complementary developmental approach targets muscle preservation during GLP‐1–induced weight loss. In the BELIEVE phase 2 trial, bimagrumab combined with semaglutide resulted in greater overall weight loss than semaglutide alone, primarily from fat, while maintaining lean and visceral fat–free mass [54]. In the EMBRAZE phase 2 study, adding apitegromab to tirzepatide yielded similar overall weight loss but preserved an additional 1.9 kg (54.9% more) of lean mass versus tirzepatide alone, improving the composition of weight change without major safety signals [55]. Together, these data illustrate emerging ‘quality of weight loss’ adjuncts rather than primary OMMs.

### 5.2 Off‐Label Multi‐Drug Regimens and Add‐On Strategies

Off‐label multidrug regimens often use GLP‐1 RAs as the base, adding traditional OMMs for patients with partial or plateaued weight loss. These strategies adapt mechanistic frameworks—central appetite, peripheral/metabolic and hedonic regulation—into practical, personalised combinations. For example, PHEN/TPM can be added to a GLP‐1 RA to enhance appetite suppression and tackle residual hunger or evening binge eating, while the GLP‐1 RA keeps satiety and glycemic control [6, 8]. Similarly, NB may be combined with GLP‐1 therapy in patients with cravings, food addiction symptoms, or cue‐driven overeating to suppress both homeostatic and hedonic drives [9, 10]. Retrospective data suggest adding NB to GLP‐1 RAs may enhance weight loss, especially in poor responders, but these findings are unconfirmed after the main study was retracted [56].

GLP‐1 RAs are also combined with SGLT‐2 inhibitors in people with T2D and obesity to extend both metabolic and cardiorenal benefits [12]. This combination produces greater weight loss than either GLP‐1 RA or SGLT‐2 inhibitor alone, with additional glycemic and metabolic benefits and maintains overall safety, aside from somewhat higher gastrointestinal adverse events [57]. While such off‐label strategies can lead to additional weight loss in select patients, evidence is limited, and they pose risks of gastrointestinal, cardiovascular and neuropsychiatric toxicity. Without robust long‐term data, they should be used cautiously in specialist settings; more rigorous evaluation of multidrug strategies is needed.

## 6 Translating Combination Pharmacotherapy Into Clinical Practice

Taken together, these data raise practical questions about when and how to deploy combinations and which research priorities should guide next‐generation regimens. Implementing combination pharmacotherapy in routine care requires aligning the mechanistic rationale with the patient's phenotype while ensuring safety and adherence to current evidence. This section outlines practical considerations for selecting combination regimens based on obesity phenotype, comorbidities, treatment response and safety constraints.

### 6.1 Current Guideline Stance and General Principles

Current guidelines generally support a stepwise, individualised approach to obesity pharmacotherapy, beginning with lifestyle intervention and, when indicated, an approved OMM, with escalation guided by treatment response, comorbidity burden, safety and access. Approved fixed‐dose combinations may be used when supported by evidence and labeling, whereas off‐label multi‐drug regimens should be approached cautiously and are best reserved for clinicians with obesity‐medicine expertise, ideally within structured follow‐up and careful monitoring [58, 59, 60]. Combination pharmacotherapy is especially justified for patients with severe obesity, associated complications, or those who do not respond well or cannot tolerate monotherapy despite good adherence. In these situations, the choice of regimen should be personalised to the patient's clinical phenotype, comorbidities, expected benefits, safety considerations and treatment burden, rather than relying on random trial‐and‐error stacking [58, 59, 60].

### 6.2 Patient Selection and Phenotyping

Clinically, combination pharmacotherapy is best reserved for patients with severe or refractory obesity, particularly when bariatric surgery is contraindicated or declined. Combination regimens are also suitable for high‐risk metabolic phenotypes, such as T2D, MASLD, or established CVD, in whom greater weight loss is expected to yield substantial downstream benefits [58, 59, 60]. They may be considered when high‐ or standard‐dose OMM monotherapy is poorly tolerated. Another indication is the presence of prominent hedonic or binge‐eating features that have not responded adequately to a single agent [58, 59, 60]. In clinical practice, combination pharmacotherapy is often used in time‐sensitive or high‐stakes settings, such as preoperative optimisation before bariatric or living kidney donor surgery. It may also be appropriate when patient expectations and psychosocial factors (e.g., an upcoming marriage or a major life event) necessitate more rapid weight loss, provided the overall risk–benefit profile remains favourable [58, 59, 60].

A useful rule of thumb is to match key clinical problems to mechanistic axes. Predominant hunger or poor satiety may favour central/hormonal satiety‐focused combinations (e.g., a sympathomimetic or TPM plus a GLP‐1 RA) [6, 8]. Combinations that engage reward and impulse‐control pathways (e.g., NB or topiramate‐based regimens) may be particularly useful for patients with prominent hedonic overeating, binge‐eating tendencies, or strong cue‐driven intake, provided psychiatric risk is carefully assessed [19, 20]. For individuals with obesity and T2D, MASLD, or high cardiovascular risk, combinations that include GLP‐1 RAs and SGLT2 inhibitors, or multi‐agonists with established cardiometabolic benefits, are most appropriate when access and tolerability allow [10, 12]. Patients with severe obesity, early onset disease, or significant weight regain risk might require earlier use of potent multi‐agonist or fixed‐dose combinations, weighing safety and adherence concerns.

### 6.3 Sequencing and Intensification Strategies

Translating the conceptual framework into practice involves deciding when to add, when to intensify, when to switch and when to de‐escalate therapy. A pragmatic, guideline‐aligned approach is to start with lifestyle intervention plus a single agent selected for comorbidities, safety profile and access; define an early response target (e.g., ≥ 5% weight loss at 3 months); and then intensify or modify treatment based on observed benefit and tolerability [58, 59, 60]. Where response is inadequate, despite good adherence, clinicians can either optimise the dose within the therapeutic range or add a second, mechanistically distinct drug, avoiding combinations with overlapping sympathomimetic effects, excessive gastrointestinal burden or compounded psychiatric risk [58, 59, 60]. Once a satisfactory plateau is reached, it is reasonable to re‐evaluate the need for all components and consider step‐down as long as weight and comorbidities are stable [58, 59, 60].

### 6.4 Monitoring and Safety in Combination Regimens

For combination regimens, monitoring should be guided by product labeling and key obesity guidelines. This includes assessing weight, blood pressure, heart rate, glycemic status, renal and hepatic function, psychiatric history and reproductive risks at baseline [58, 59, 60]. Early follow‐up (e.g., every 4–12 weeks) should be planned to assess weight trends, tolerability, adherence and any new adverse effects, including gastrointestinal symptoms, dehydration, warning signs of gallbladder or pancreatic adverse events, cardiovascular changes, mood or sleep disturbances and cognitive problems [58, 59, 60]. Reproductive counselling and, when appropriate, baseline and periodic pregnancy testing are essential for women of childbearing potential who are using agents with teratogenic risk, such as TPM. Dose adjustments should be made one component at a time, and clinicians should consider regimen simplification or step‐down once a stable, clinically meaningful response is achieved [58, 59, 60].

### 6.5 Treatment Discontinuation and Weight Regain

Weight trajectories following dose reduction or discontinuation of combination therapy require careful monitoring. In obesity pharmacotherapy trials, weight loss is typically attenuated or partially reversed after medication discontinuation, even when lifestyle support continues [61, 62]. Similar patterns are expected with multi‐drug and multi‐receptor regimens. Clinicians should plan long‐term follow‐up, anticipate potential weight regain, and incorporate relapse‐prevention strategies, including ongoing behavioural support and, when appropriate, reinitiation or adjustment of pharmacotherapy. When de‐escalating or discontinuing treatment, expectations for likely weight changes should be discussed explicitly so that patients do not interpret regain as personal failure but as a common feature of chronic obesity care that may necessitate renewed or modified therapy.

**FIGURE 4:**
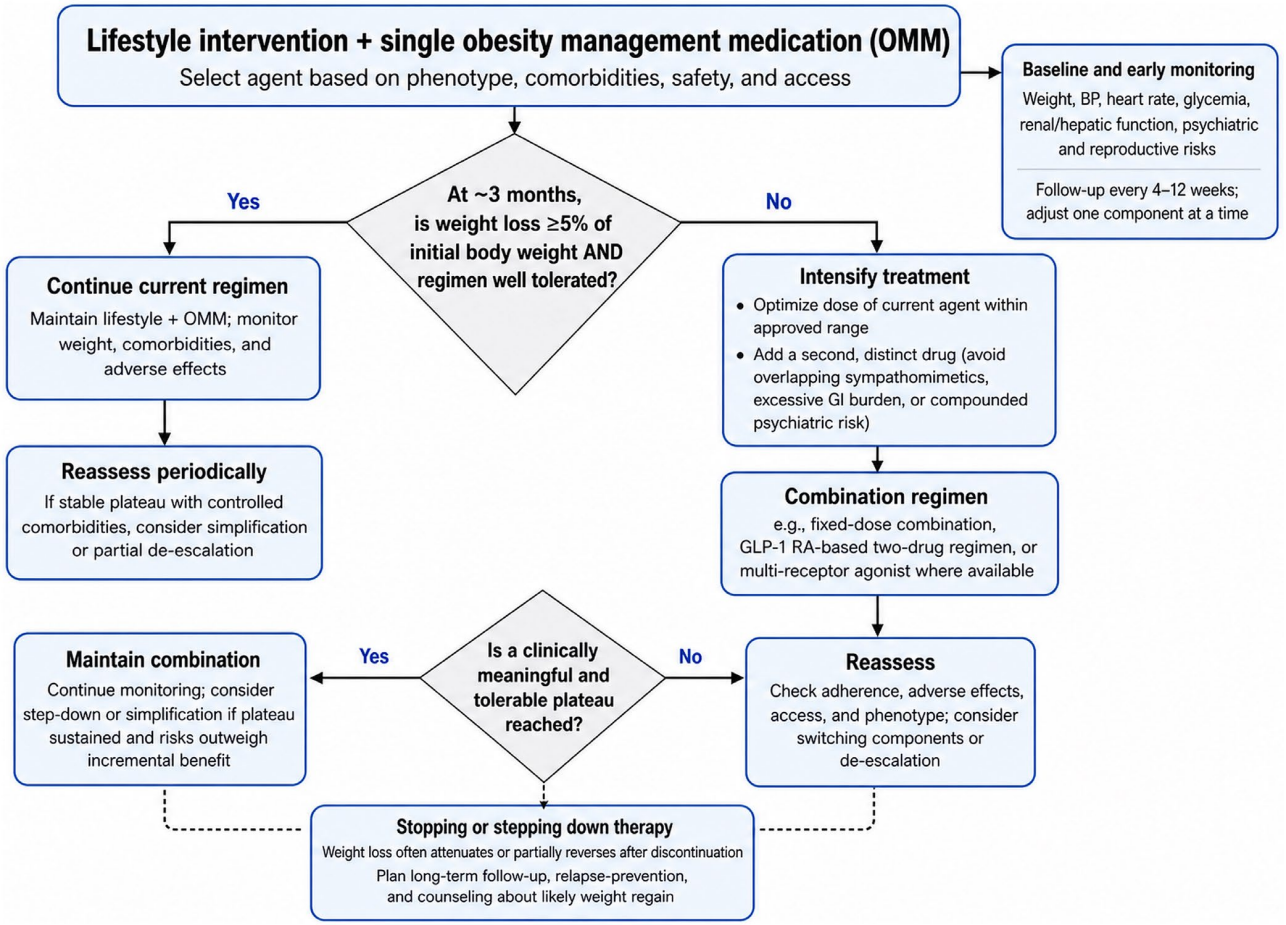
Conceptual treatment algorithm for lifestyle intervention and combination pharmacotherapy in obesity, illustrating initial single‐agent therapy, criteria for intensification, monitoring and safety considerations and planning for de‐escalation, discontinuation and management of weight regain. OMM, obesity management medication.

As summarised in Figure 4, baseline monitoring, early reassessment and long‐term follow‐up inform decisions on intensification, de‐escalation and discontinuation.

### 6.6 Cost, Access and Adherence

Beyond efficacy, the long‐term impact of combination pharmacotherapy will depend on sustainable access to medications, insurance or public‐sector coverage for high‐cost agents, and strategies to support adherence to complex multidrug regimens in everyday practice. These practical issues often determine whether patients can sustain combination pharmacotherapy over time [58, 59, 60, 63]. Practical measures to support adherence include:

- Simplifying regimens wherever possible (e.g., weekly injections rather than daily if options exist; once‐ or twice‐daily oral dosing instead of multiple daily doses).
- Matching dosing schedules to existing routines (e.g., morning dosing for agents that may cause insomnia).
- Providing written, detailed instructions about what to do if doses are missed or adverse effects occur
- Setting realistic expectations about the rate and extent of weight loss, emphasising that a slower, steady approach with high consistency is better in the long run than rapid weight loss interrupted by side effects or costs

For payers, decisions about funding high‐cost agents will likely be based on data beyond weight loss, including cardiovascular and renal outcomes, quality‐of‐life outcomes and cost‐effectiveness analyses.

### 6.7 Strengths and Limitations of Current Evidence

The current evidence base for combination pharmacotherapy in obesity has several strengths. Approved fixed‐dose combinations are supported by randomised controlled trial data, add‐on approaches are increasingly informed by clinical experience, and rapid advances in multi‐receptor agonist development have expanded the therapeutic landscape. Collectively, these data support the biologic rationale that targeting complementary pathways may improve weight loss and metabolic outcomes beyond monotherapy in selected patients. In addition, emerging efforts to align treatment choice with obesity phenotype and comorbidity profile may improve the clinical applicability of combination strategies.

However, the quality and maturity of evidence remain uneven across treatment categories, with far more robust support for approved combinations than for off‐label multidrug regimens and many emerging agents. Much of the literature consists of relatively short trials in selected populations, while observational and preclinical data should be interpreted more cautiously than randomised evidence. Important gaps remain in long‐term safety, treatment discontinuation, weight regain after cessation, cost‐effectiveness, optimal sequencing or de‐escalation and head‐to‐head comparative‐effectiveness studies. Accordingly, investigational and off‐label strategies should not be overinterpreted or generalised to routine practice without further high‐quality data and specialist oversight.

## 7 Future Directions and Research Agenda

### 7.1 Unanswered Clinical Questions

Key uncertainties persist about the timing for introducing combination pharmacotherapy, identifying who benefits most and the longevity of these benefits. Specifically, it is still uncertain whether combinations should be used only after monotherapy fails or earlier in certain high‐risk groups, such as those with severe obesity, obesity with T2D, MASLD, CVD, or prominent hedonic eating [58, 59, 60]. Future research should explore how different combinations affect outcomes beyond weight loss, including glycaemia, liver disease, kidney outcomes, cardiovascular events, physical function and body composition. Additionally, the long‐term durability of these benefits and the potential for relapse after stopping treatment remain insufficiently understood, especially for multi‐drug regimens and newer multi‐agonist peptides.

### 7.2 Trial Design Priorities

Advancing progress requires shifting from short‐term placebo‐controlled efficacy trials to large, long‐term randomised studies and pragmatic comparative‐effectiveness research. These investigations should compare optimised monotherapy with fixed‐dose combinations, add‐on regimens and multi‐receptor agonists, and include adequate follow‐up to assess maintenance, relapse, safety and treatment burden. Factorial, adaptive and head‐to‐head designs can help distinguish synergistic from redundant combinations and assess the relative potency of intensive pharmacotherapy versus bariatric and endoscopic options.

### 7.3 Mechanistic and Translational Research

Mechanistic research helps to better select drugs and patients by identifying biological and behavioural markers associated with response, intolerance, or relapse. Methods such as multi‐omics, neurobehavioral profiling and digital monitoring help match treatments with important obesity drivers, such as impaired satiety, hedonic overeating, or metabolic dysfunction [13, 19, 20, 64]. Additionally, these methods may facilitate advanced strategies to improve the quality of weight loss, including the preservation of lean mass and physical function [54, 55].

### 7.4 Regulatory, Ethical and Health‐System Priorities

Regulatory, ethical and health‐system issues will strongly shape the trajectory of combination pharmacotherapy for obesity [58, 59, 60]. Approval of fixed‐dose products and unimolecular multiagonists typically requires evidence of superiority over optimised individual components, clear justification of dose ratios and long‐term safety data covering cardiovascular, renal and mortality outcomes [58, 59, 60]. Meanwhile, off‐label multidrug ‘stacks’, compounded formulations and telehealth‐driven regimens raise concerns about informed consent, risk–benefit documentation, monitoring intensity and equitable access, particularly when costs are high and coverage varies [58, 59, 60, 64]. Innovation and standardisation must be balanced by health systems with complex regimens delivered through structured, evidence‐based pathways. Future research should also focus on real‐world challenges like medication supply, reimbursement policies and adherence to multi‐drug or injectable regimens, as these factors will ultimately influence the broader implementation of these therapies beyond specialist centres.

## 8 Conclusion

Combination pharmacotherapy is increasingly relevant in obesity care because many patients have overlapping disturbances in appetite, hedonic drive and metabolic adaptation rather than a single dominant mechanism. In current practice, the strongest evidence supports the use of approved fixed‐dose combinations such as PHEN/TPM ER and NB, which show that targeting complementary pathways can improve weight loss beyond monotherapy, though with a greater need for monitoring for cardiovascular, neuropsychiatric and other adverse effects. Emerging multi‐receptor agonists and investigational muscle‐preserving adjuncts may further expand treatment options and improve not only the magnitude but also the quality of weight loss. The available evidence, primarily from early‐phase trials, shows promise but is inconsistent in quality. Substantial gaps exist in long‐term safety, durability, effectiveness and real‐world use that must be addressed before recommending wider use of complex combination regimens. For clinicians, the key challenge is not simply choosing the most potent regimen but matching therapy to phenotype, comorbidity profile, tolerability, access and patient preference. Off‐label multidrug combinations may be reasonable for selected patients with severe or refractory obesity, but should be used cautiously, with a clear mechanistic rationale and structured follow‐up. Until longer‐term comparative and outcome data are available, combination pharmacotherapy should be used in a stepwise, individualised and safety‐conscious manner rather than driven solely by weight‐loss efficacy.

## Author Contributions

**Arnav Kalra:** writing – original draft, resources, investigation, data curation, visualization. **Robin Maskey:** supervision, writing – review and editing, resources, visualization, investigation. **Nitin Kapoor:** writing – review and editing, resources, investigation, methodology, validation. **Deep Dutta:** writing – review and editing, resources, validation, investigation, data curation. **Kunal Mahajan:** writing – review and editing, resources, visualization, investigation, data curation. **Syed Abbas Raza:** writing – review and editing, resources, supervision, project administration, investigation. **A. B. M. Kamrul‐Hasan:** conceptualization, writing – original draft, writing – review and editing, resources, project administration, visualization, supervision, data curation, investigation, methodology.

## Funding

The authors have nothing to report.

## Conflicts of Interest

The authors declare no conflicts of interest.

## Supporting information


**Table S1:** Multi‐receptor peptide agonist obesity management medications under development: key clinical outcomes.

## Data Availability

The authors have nothing to report.
