# Supplementary material for: Combination Pharmacotherapies for Obesity—Current Evidence and Future Directions
Source: Endocrinol Diabetes Metab. 2026 Aug 11;9(5):e70307. doi: 10.1002/edm2.70307 (PMC13462310; doi:10.1002/edm2.70307)
Supplement: Supplementary file 1 [file EDM2-9-e70307-s001.docx]

**Supplementary Table 1. Multi‑receptor peptide agonist obesity management medications under development: key clinical outcomes**

| **Agent, Developer** | **Receptor targets** | **Approximate mean placebo-ajusted weight loss vs baseline** | **Selected metabolic outcomes** | **Major adverse events** | **Development phase / status in obesity** |
| --- | --- | --- | --- | --- | --- |
| Retatrutide (LY3437943), Eli Lilly [48] | Triple GLP-1/GIP/GCG receptor agonist | Phase 2 trial: -22.1% placebo‑adjusted mean weight loss with 12 mg at 48 weeks | Improved fasting glucose, insulin sensitivity, lipids and BP; in T2D cohorts, ~16-17% loss with ~2% HbA1c reduction | GI AEs most frequent; dose‑dependent increase in heart rate (~6–7 beats/min) and reports of mild arrhythmias; long‑term cardiovascular outcome data pending | Phase 2 completed;  Phase 3 TRIUMPH/TRANSCEND obesity and T2D programmes ongoing, with topline phase 3 data reporting mean weight loss up to ~24–28% in obesity cohorts |
| Survodutide (BI 456906), Boehringer Ingelheim/Novo Nordisk [49] | Dual GLP-1/GCG receptor agonist | Phase 2 trial: at 46 weeks, placebo‑adjusted mean weight loss-  0.6 mg -3.4%,  2.4 mg -9.7%,  3.6 mg 10.4%,  4.8 mg -12.1% | HbA1c reduction in T2D, improved triglycerides and liver fat content | GI adverse events (nausea, vomiting, diarrhoea) similar to GLP‑1 RAs; dose‑dependent heart‑rate increases observed; long‑term cardiovascular outcome data pending. | Phase 2 obesity and MASLD trials completed; phase 3 underway SYNCHRONIZE |
| Pemvidutide (ALT-801), Altimmune [50] | Dual GLP-1/GCG receptor agonist | Phase 2 MOMENTUM trial: at 46 weeks, placebo‑adjusted mean weight loss-  1.2 mg -8.1%,  1.8 mg -9.0%,  2.4 mg -13.4% | Reduced liver fat fraction and markers of steatohepatitis, improved glycaemia and lipids | GI adverse events most common (nausea, vomiting, diarrhoea); GLP‑1‑like tolerability profile; low rates of serious AEs in phase 2 | Phase 2 obesity and MASH trials completed; phase 2 MOMENTUM obesity trial ongoing, with phase 3 planning announced |
| Maridebart cafraglutide (AMG 133, “MariTide”), Amgen [51] | Long-acting peptide–antibody conjugate – GLP-1 RA and GIP-receptor antagonist | Phase 2 trial:  placebo-adjusted weight loss at 52 weeks with once-monthly dosing -11.1 to -13.8% | Improvements in fasting glucose, insulin resistance indices, triglycerides and non‑HDL cholesterol; modest reductions in systolic blood pressure and inflammatory markers vs placebo | GI adverse events common, less frequent with dose escalation and a lower starting dose. | Phase 2 trials complete. Phase 3 MARITIME clinical trials ongoing |
| CagriSema (Cagrilintide + semaglutide); Novo Nordisk [52] | Amylin analogue + GLP-1 RA (functional dual agonism) | REDEFINE 1 trial:  -17.4% placebo-ajusted weight loss at 68 weeks, and exceeding semaglutide alone (by -11.9% more weight loss) at similar doses | Additional HbA1c and fasting glucose reductions vs semaglutide alone, greater reductions in energy intake and improved lipids | Higher rates of nausea and injection‑site reactions vs semaglutide but similar overall | Phase 3 REDEFINE programme completed; regulatory submissions (including US FDA) underway for chronic weight management and T2D |
| Zenagamtide (Amycretin), Novo Nordisk [53] | Unimolecular GLP-1/amylin/ calcitonin receptor agonist | Phase 2 data for SC: placebo at week 36  placebo-ajusted weight loss-  60 mg -23.2% (at 36 weeks),  20 mg -23.9% (at 36 weeks),  5 mg -18.6% (at 28 weeks),  1.25 mg -11.7% (at 20 weeks) | Reductions in waist circumference and visceral adiposity indices; improvements in fasting glucose, HbA1c and lipid profile (triglycerides and LDL cholesterol), with small decreases in blood pressure | Predominantly gastrointestinal (nausea, vomiting, diarrhoea), mild to moderate. | Phase 2 trials ongoing for both oral and SC. |

AE, Adverse events; GCG, glucagon; GLP-1, Glucagon-like peptide-1; GI, Gastrointestinal; GIP, Glucose-dependent insulinotropic polypeptide; HbA1c, Glycated hemoglobin; LDL, Low-density lipoprotein; MASH, Metabolic dysfunction-associated steatohepatitis; RA, Receptor agonist; SC, Subcutaneous; T2D, Type 2 diabetes; US FDA, United States Food and Drug Administration.
